# Reconstruction of metabolic module with improved promoter strength increases the productivity of 2-phenylethanol in *Saccharomyces cerevisiae*

**DOI:** 10.1186/s12934-018-0907-x

**Published:** 2018-04-11

**Authors:** Zhaoyue Wang, Mingyue Jiang, Xuena Guo, Zhaozheng Liu, Xiuping He

**Affiliations:** 1 CAS Key Laboratory of Microbial Physiological and Metabolic Engineering, State Key Laboratory of Mycology, Institute of Microbiology, Chinese Academy of Sciences, Beijing, China; 2 College of Life Sciences, University of Chinese Academy of Sciences, Beijing, China; 3 Beijing No. 2 Middle School, Beijing, China

**Keywords:** *Saccharomyces cerevisiae*, *GAP1 *+ *ARO8 *+ *ARO10 *+ *ADH2 *+ *GDH2*, 2-phenylethanol, Promoter strategy, Metabolic module, Fermentation optimization

## Abstract

**Background:**

2-phenylethanol (2-PE) is an important aromatic compound with a lovely rose-like scent. *Saccharomyces cerevisiae* is a desirable microbe for 2-PE production but its natural yield is not high, and one or two crucial genes’ over-expression in *S. cerevisiae* did not improve 2-PE greatly.

**Results:**

A new metabolic module was established here, in which, permease Gap1p for l-phenylalanine transportation, catalytic enzymes Aro8p, Aro10p and Adh2p in Ehrlich pathway respectively responsible for transamination, decarboxylation and reduction were assembled, besides, glutamate dehydrogenase Gdh2p was harbored for re-supplying another substrate 2-oxoglutarate, relieving product glutamate repression and regenerating cofactor NADH. Due to different promoter strengths, *GAP1*, *ARO8*, *ARO9*, *ARO10*, *ADH2* and *GDH2* in the new modularized YS58(G1-A8-A10-A2)-GDH strain enhanced 11.6-, 15.4-, 3.6-, 17.7-, 12.4- and 7.5-folds respectively, and crucial enzyme activities of aromatic aminotransferases and phenylpyruvate decarboxylase were 4.8- and 7-folds respectively higher than that of the control.

**Conclusions:**

Under the optimum medium and cell density, YS58(G1-A8-A10-A2)-GDH presented efficient 2-PE synthesis ability with ~ 6.3 g L^−1^ of 2-PE titer in 5-L fermenter reaching 95% of conversation ratio. Under fed-batch fermentation, 2-PE productivity at 24 h increased 29% than that of single-batch fermentation. Metabolic modularization with promoter strategy provides a new prospective for efficient 2-PE production.

## Background

2-Phenylethanol (2-PE) is a kind of aromatic alcohol with a rose-like fragrance. It naturally exists in the essential oils of many plants and flowers, and is also a natural product of some fermented foods [1, 2]. 2-PE has a wide range of applications in the perfumery, cosmetics and fermented food industries for its lovely scent [3], and also can be used as a substrate for the synthesis of other flavors or pharmaceutical compounds [4]. Compared to the traditional methods such as chemical synthesis and natural extraction, production of 2-PE by microbial fermentation is a breakthrough which is efficient and economic [5]. As a significant eukaryotic model organism with high tolerance towards many stress factors, *Saccharomyces cerevisiae* is a desirable strain for 2-PE production [4, 6].

**Fig. 1:**
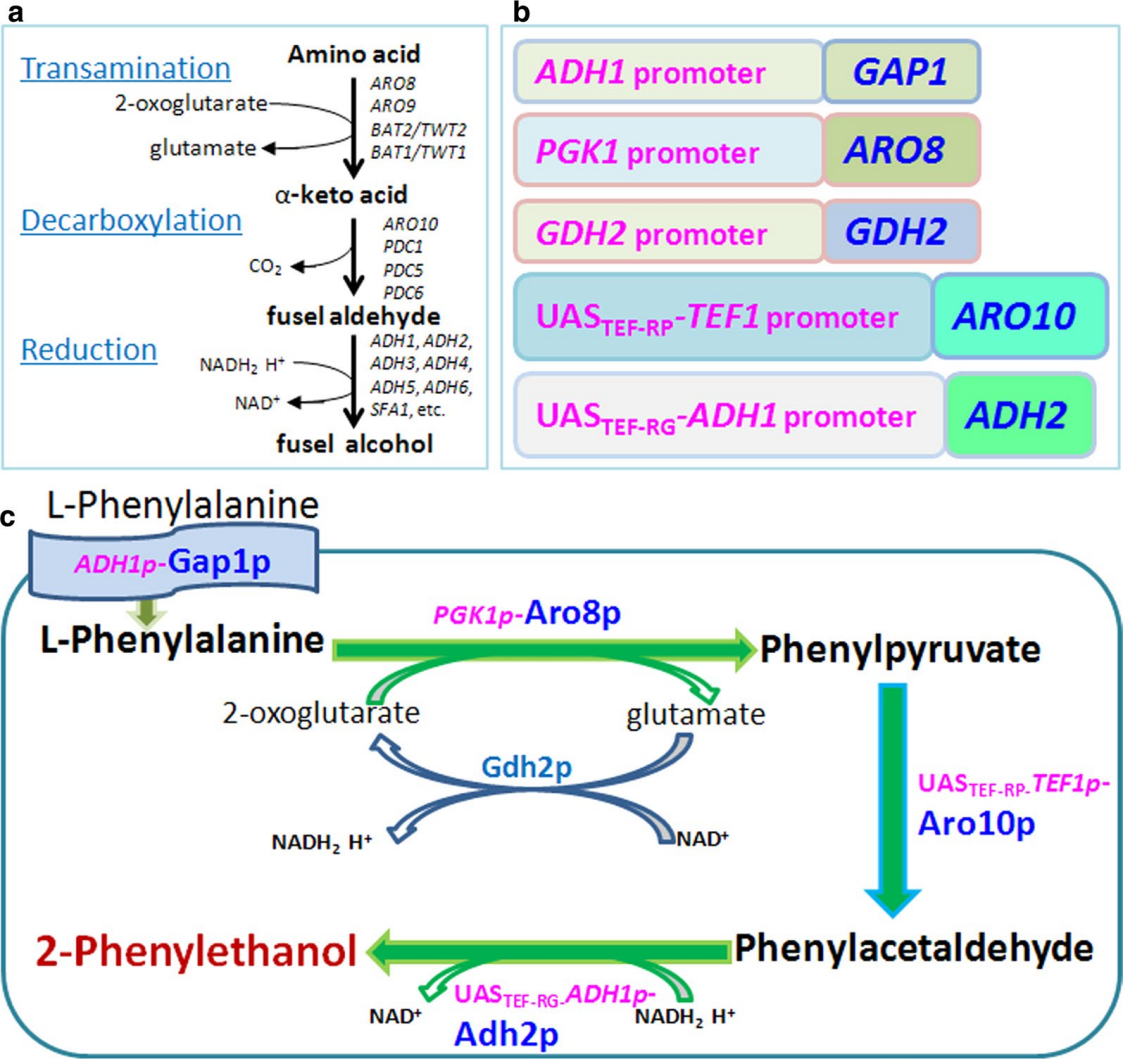
Ehrlich pathway and diagram of new expression module for 2-PE synthesis under different promoter strengths. **a** Ehrlich pathway in yeasts. **b** Modified cassettes with different promoter strengths. **c** Diagram of new expression module

Two principal pathways exist in *S. cerevisiae* for 2-PE synthesis. Shikimate pathway is a long de novo synthesis pathway with multiple-branches and a variety of feedback inhibitions, whose 2-PE production is very low. When l-phenylalanine (l-Phe) is used as the sole nitrogen source in the medium, 2-PE can be synthesized via Ehrlich pathway (Fig. 1a), in which aromatic aminotransferases (Aro8p and Aro9p), phenylpyruvate decarboxylase (Aro10p) and alcohol dehydrogenases mainly participate [4, 7]. *S. cerevisiae* can adjust the physiological metabolism according to the content and quality of nitrogen source [8]. Gap1p, a general amino acids permease [9], can be induced to express and transport l-Phe into yeast cells [9, 10]. In Ehrlich pathway, transamination reaction catalyzed by Aro8p and Aro9p [11, 12] and decarboxylation mainly catalyzed by Aro10p [13] are very crucial for 2-PE synthesis. Aro8p expression is regulated by general control of amino acid biosynthesis, and Aro9p and Aro10p are induced to express by high concentrations of aromatic amino acids [14–17]. Enhancing Aro8p, Aro9p or Aro10p expression could promote 2-PE production [10, 17, 18].

Some regulatory factors for Ehrlich pathway have been partly elucidated in recent years. Aro80p, a zinc finger transcriptional activator in the Zn2Cys6 family, can accept the inducing signals from aromatic amino acids and bind to the repeat sequences on promoters of *ARO9* and *ARO10* to activate them expressing [11, 14]. Expression of *ARO9* and *ARO10* can also be activated by Gat1p and Gln3p [10, 19]. Gln3p and Gat1p are GATA-family zinc finger transcriptional activators regulated by global nitrogen quality control known as nitrogen catabolite repression (NCR) [19–23]. In a good nitrogen condition, Gln3p and Gat1p are sequestered in the cytosol. When the nitrogen source is poor, Gln3p and Gat1p translocate to the nucleus, activating the NCR-sensitive genes such as *ARO9* and *ARO10* as well as *GAP1* with GATA-sequences motif resulting in 2-PE synthesis increased [8, 10, 24]. Wuster and Babu [25] ever predicted that transcription factors Cat8p and Mig1p were related to some genes’ expression responsible for aromatic alcohol synthesis. Cat8p, a zinc cluster transcriptional activator, can bind to the carbon source-responsive element (CSRE) motifs to make key gluconeogenic enzymes derepressed and activated [26–30]. Mig1p is a transcription factor involved in glucose repression [31, 32]. In recent research, Cat8p and Mig1p were proved to be regulatory factors of 2-PE synthesis. Cat8p over-expression or Mig1p deletion could positively regulate transaminase and phenylpyruvate decarboxylase activities leading 2-PE synthesis enhanced [18].

The methods for improving 2-PE yield of yeast mainly include traditional breeding methods, fermentation conditions optimization and in situ 2-PE removal [33–39]. With the elucidation of regulatory mechanism of 2-PE biosynthesis, metabolic engineering strains were constructed, whose 2-PE synthetic ability were improved in different degrees [10, 17, 18]. Currently, the engineering strains were mainly operated by over-expressing one or two genes of transaminases, phenylpyruvate decarboxylase, alcohol dehydrogenases, transcription factors or permease [10, 17, 18, 40, 41], however, these genetically modification did not improve 2-PE yield greatly, probably due to the limitation of substrate transportation or unfluent reactions in Ehrlich pathway or some repression.

Up to now, no globally enhanced synthetic net including substrate supplement and all reaction steps has been considered and investigated. In this study, a new metabolic module was established, in which, Gap1p for l-Phe transportation and three enzymes respectively responsible for transamination, decarboxylation and reduction were assembled, besides, glutamate dehydrogenase was also harbored aiming for relieving the repression of glutamate and re-supplying another substrate 2-oxoglutarate, in the meantime, cofactor NADH was regenerated. Promoter strategy was adopted in this work. The genes in the new module cassette were expressed under different promoter strengths. Based on these modifications, the new modularized *S. cerevisiae* presented fluent and high efficient 2-PE synthesis ability.

## Results and discussion

### New metabolic module design with different promoter strengths

Natural 2-PE synthesized in *S. cerevisiae* is low, over-expression of one or two crucial genes up-regulated 2-PE synthesis via Ehrlich pathway but did not improve 2-PE yield greatly [10, 17, 18, 40], therefore, new strategy should be considered. Up to now, most of the regulatory transcription factors such as Aro80p, Gat1p, Gln3p and Cat8p were reported to promote 2-PE synthesis by targeting at genes of amino acid permease, crucial enzyme aromatic aminotransferase and phenylpyruvate decarboxylase [10, 18, 24], hence, it is appropriate and necessary in the new design to include relative elements responsible for substrate transportation and crucial reactions. Module balance should also be considered, thus, non-rate-limiting alcohol dehydrogenase was contained. The by-product glutamate produced from the first transamination reaction is a kind of good nitrogen for yeast probably repressing Ehrlich pathway to synthesize 2-PE, in order to relieve the repression and make the whole pathway fluently, non-pathway-specific glutamate dehydrogenase Gdh2p was further designed to the metabolic module. Harboring Gdh2p would lead cofactor NADH regeneration making the third reduction in Ehrlich pathway more fluent (Fig. 1a, c). The mechanism by which 2-PE is exported from the cells into the culture medium remains unknown [7]. It is conceivable that export occurs by simple passive diffusion across the lipid bilayer [42], hence, no relative gene for exportation was designed in our module.

Promoter strength is significant for gene expression, different design among target genes according to their specificity and significance to the whole metabolism should be favorable for 2-PE synthesis. *ADH1p*, *PGK1p* and *TEF1p* are three constitutive strong promoters respectively from alcohol dehydrogenase I, 3-phosphoglycerate kinase and translational elongation factor EF-1 alpha. In our lab, constitutive and tunable promoter library with strength variation of 1–19 folds has been established using synthetic hybrid promoter approach, in which enhancer elements such as transcription factors Rap1p, Gcr1p and/or Sgp1p binding sites and upstream activation sequences (UAS) were included [43, 44]. UAS_TEF-RP-_*TEF1p*, one member in our promoter library derived from *TEF1p* is stronger than *TEF1p*. Similarly, UAS_TEF-RG-_*ADH1p* derived from *ADH1p* is stronger than *ADH1p*. Permease Gap1p is essential for substrate transportation, constitutive promoter *ADH1p* was used in it. The stronger promoter *PGK1p* was designed to harbor in the upstream of aromatic aminotransferase Aro8p for catalyzing the first pathway-specific reaction in Ehrlich pathway. Phenylpyruvate decarboxylase (Aro10p) is the rate-limiting enzyme, the strongest UAS_TEF-RP-_*TEF1p* was used to regulate it (Fig. 1b). In the third step of reduction reaction, one of the alcohol dehydrogenases ADHII is a glucose-repressible enzyme, for speeding up reduction step, constitutive and stronger promoter UAS_TEF-RG-_*ADH1p* was chosen to put to the upstream of *ADH2* (Fig. 1b). Glutamate dehydrogenase Gdh2p was not the pathway-specific enzyme for 2-PE metabolism, it was designed to use its own promoter. In the new module, there are gradually increased promoting strengths to regulate gene expressing responsible for relative enzymes from substrate transportation to most important decarboxylation reaction. l-Phe should be absorbed fast by yeast cells, transamination and decarboxylation ought to be boosted. Over-expressed Gdh2p is probably helpful for removing glutamate repression and for increasing substrate 2-oxoglutarate. NADH regeneration from reaction catalyzed by Gdh2p and enhanced ADHII would accelerate reduction reaction to synthesize 2-PE efficiently. The global modularized up-regulation of enzyme activities was supposed to improve 2-PE synthesis ability in a large degree (Fig. 1c).

### Construction of expression cassettes, screening of engineering yeast strains and verification

**Fig. 2:**
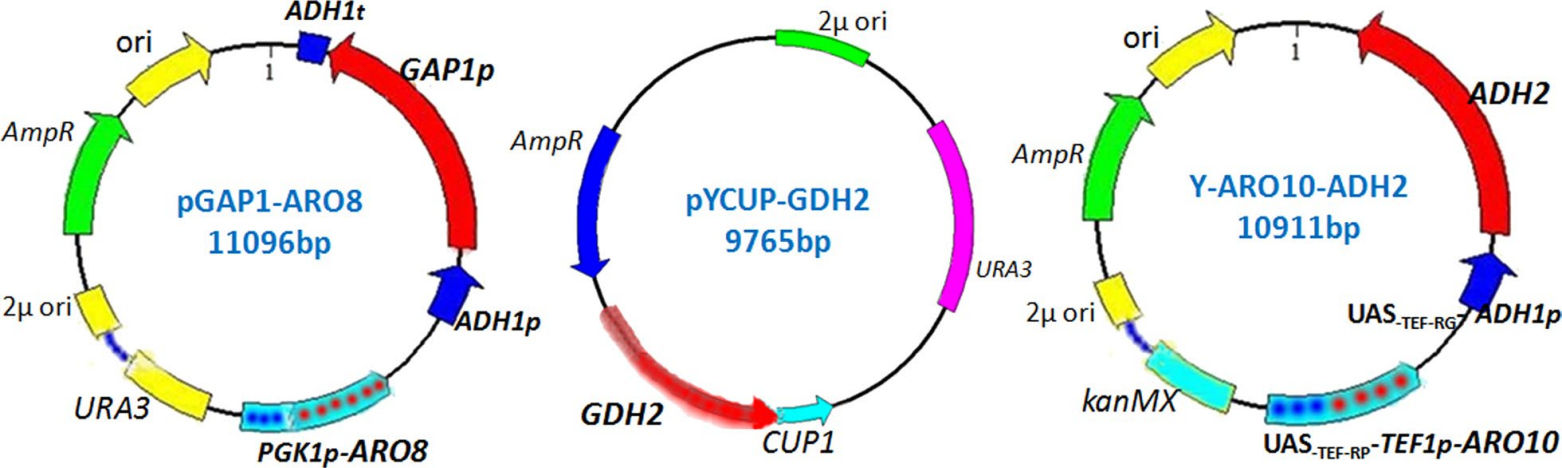
Plasmid maps of *GAP1 *+ *ARO8*, *GDH2* and *ARO10 *+ *ADH2* expression cassettes. Plasmid pGAP1-ARO8 contained *GAP1* and *ARO8* expression cassettes with *ADH1p* and *PGK1p* promoter respectively and *URA3* as selection marker; plasmid pYCUP-GDH2 contained *GDH2* expression cassette with its own promoter and *CUP1* as selection marker; Plasmid Y-ARO10-ADH2 contained *ARO10* and *ADH2* expression cassettes with UAS_-TEF-RP_-*TEF1* and UAS_-TEF-RG_-*ADH1* promoter respectively and *kanMX* as selection marker

Based on above design, a series of recombined plasmids were constructed. In the combined plasmid pGAP1-ARO8, expression cassettes *ADH1p*-*GAP1* and *PGK1p*-*ARO8* were harbored (Fig. 2). In plasmid Y-ARO10-ADH2, expression cassettes UAS_-TEF-RP_-*TEF1p*-*ARO10* and UAS_-TEF-RG_-*ADH1p*-*ADH2* were contained (Fig. 2). Plasmid pYCUP-GDH2 carried *GDH2* expression cassette (Fig. 2). The sequencing results indicated that the coding sequences of the above genes were the same as model strain S288C in NCBI.

In our previous work, some recombined strains with single gene over-expressing have been constructed such as YS58(ARO8-58), YS58(ARO10-58) and YS58(YEpAP58) harboring *ARO8*, *ARO10* and *GAP1* plasmid respectively [10, 18]. In this study, not only new single gene expressing strains YS58(ADH2) and YS58(GDH2) were selected, a series of co-expressing strains were also obtained, in which, strain YS58(ARO8 + ARO10) carrying *ARO8 *+ *ARO10*, YS58(A8-A10-A2) harboring *ARO8 *+ *ARO10 *+ *ADH2*, YS58(G1-A8-A10-A2) containing *GAP1 *+ *ARO8 *+ *ARO10 *+ *ADH2* and YS58(G1-A8-A10-A2)-GDH including *GAP1 *+ *ARO8 *+ *ARO10 *+ *ADH2 *+ *GDH2* expression cassettes. Successful transformants were characterized by PCR analysis. In order to make the experimental results convincing, all the recombined strains were cultivated on selective and non-selective media alternately for 20 generations, stable engineering strains were recollected and verified again to be right.

### Comparison of transcription levels, enzyme activities, cell growths and 2-PE titers

**Fig. 3:**
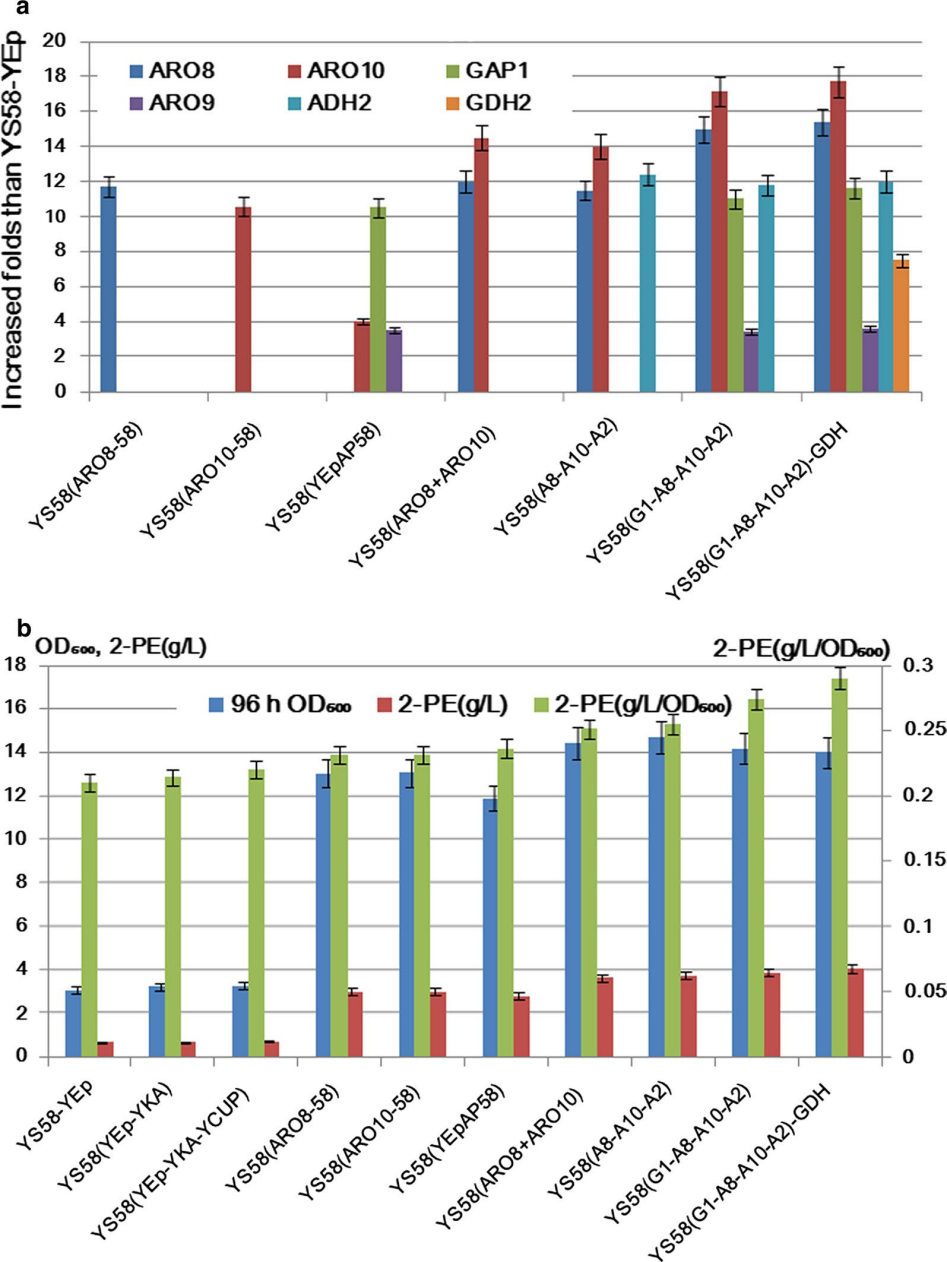
Comparison of transcription levels of over-expressed genes, cell growths and 2-PE titers among engineering strains. **a** Enhanced folds of gene transcription levels in transformants. **b** Cell growths, 2-PE titers and specific production of 2-PE of engineering strains and control strains

Different promoting strengths in engineering strains were reflected from both mRNA levels and enzyme activities. mRNA levels of engineering strains and their control strains were analyzed by qRT-PCR, the results showed that three control strains YS58-YEp, YS58(YEp-YKA) and YS58(YEp-YKA-YCUP) showed similar expression levels on test genes. Gene expression and 2-PE titers of strains YS58(ADH2) and YS58(GDH2) were analyzed, but the enhanced expression levels of *ADH2* and *GDH2* did not promote 2-PE synthesis (data not shown), probably owing to non-specific characteristics of Adh2p and Gdh2p to Ehrlich pathway. However, over-expression of these two genes was helpful for 2-PE synthesis in modularized strain (Fig. 3b), whose positive roles could be reflected from increased 2-PE yield in *ARO8 *+ *ARO10 *+ *ADH2* co-expression strain YS58(A8-A10-A2) than that in strain YS58(ARO8 + ARO10), and also reflected from enhanced 2-PE titer in *GAP1 *+ *ARO8 *+ *ARO10 *+ *ADH2 *+ *GDH2* co-expression strain YS58(G1-A8-A10-A2)-GDH than that in strain YS58(G1-A8-A10-A2) with *GAP1 *+ *ARO8 *+ *ARO10 *+ *ADH2* co-expression (Fig. 3b). The synergism of constitutively expression Adh2p in our module strain for 2-PE synthesis was in accordance with that of *ARO10 *+ *ADH2* co-expression strain reported by Shen et al. [40]. Compared to control strains, single gene over-expressing strains YS58(ARO8-58), YS58(ARO10-58) and YS58(YEpAP58) presented enhanced expression of *ARO8*, *ARO10*, *GAP1 *+ *ARO9 *+ *ARO10* respectively (Fig. 3a), similar to that in our previous reports [10, 18]; two to four genes over-expressing strains YS58(ARO8 + ARO10), YS58(A8-A10-A2) or YS58(G1-A8-A10-A2) showed respectively *ARO8 *+ *ARO10*, *ARO8 *+ *ARO10 *+ *ADH2* and *GAP1 *+ *ARO8 *+ *ARO9 *+ *ARO10 *+ *ADH2* increase (Fig. 3a). Differences of promoter strengths were also displayed, for example, expression of *ARO10* with promoter UAS_-TEF-RP_-*TEF1p* in YS58(ARO8 + ARO10) increased 14.5-folds than that of control, while *ARO10* with promoter *ADH1p* in YS58(ARO10-58) increased 10.6-folds than that of control, indicating that promoting force of UAS_-TEF-RP_-*TEF1p* was stronger than that of *ADH1p* (Fig. 3a). Among these strains, YS58(G1-A8-A10-A2)-GDH was our objective strain which harbored the whole metabolic module, the results of qRT-PCR indicated that all the designed five genes presented high expression levels. In addition, mRNA level of *ARO9* increased owing to regulation of Gap1p. Compared to the control strain, expression levels of *GAP1*, *ARO8*, *ARO9*, *ARO10*, *ADH2* and *GDH2* enhanced 11.6-, 15.4-, 3.6-, 17.7-, 12.4- and 7.5-folds respectively (Fig. 3a).

Analysis of enzyme activities indicated that the most crucial enzymes aromatic aminotransferases and phenylpyruvate decarboxylase in the module strain YS58(G1-A8-A10-A2)-GDH were respectively 4.8- and 7-folds higher than that of the control strain (Table 1), reflecting that increased RNA levels resulted in increased enzyme proteins’ level. Since *GAP1* over-expression could up-regulate mRNA level of *ARO9* [10], increased activities of aminotransferases in the module strain should result not only from over-expressed *ARO8* but also from *ARO9* up-regulated by *GAP1* (Fig. 3a and Table 1). Enhanced phenylpyruvate decarboxylase should be the reflection of *ARO10* increase that was led by both the strongest promoter in the module and *GAP1* regulation on original *ARO10*. Increase of the two crucial enzyme activities were accordance with that of their mRNA levels (Fig. 3a and Table 1). Cell growths of control strains YS58-YEp, YS58(YEp-YKA) and YS58(YEp-YKA-YCUP) were similar, but all the engineering strains grew faster than control strains (Fig. 3b), which might result from the acceleration of utilization of l-Phe nitrogen source [18]. The results of 2-PE analysis in tube fermentation indicated that with the number of over-expressed genes being added, 2-PE titers increased gradually. Among these strains, module strain YS58(G1-A8-A10-A2)-GDH produced the highest 2-PE titer compared to other strains, 5.65-folds higher than that of the control strain YS58(YEp-YKA-YCUP) (Fig. 3b). Considering that the raised biomass also contributed to total 2-PE concentration (g L^−1^) increase, specific production of 2PE (g/L/OD_600_) was also compared. The results showed that single gene expression such as *ARO8*, *ARO10* or *GAP1* up-regulated the yeast 2-PE (g/L/OD_600_) level 10–12%, two (*ARO8 *+ *ARO10*) or three (*ARO8 *+ *ARO10 *+ *ADH2*) genes’ expression promoted yeast cells to synthesize 2-PE 17–19% higher than the host, four (*GAP1 *+ *ARO8 *+ *ARO10 *+ *ADH2*) and five (*GAP1 *+ *ARO8 *+ *ARO10 *+ *ADH2 *+ *GDH2*) genes’ over-expression respectively led to 28 and 32% increase of 2-PE production than the control strain (Fig. 3b). The designed module strain with five genes presented the highest 2-PE synthesis ability, it was chosen for further test.

**Table 1:** Activities of crucial enzymes in different yeast strains

Strains^a^	Enzyme activities (IU mg^−1^ protein)**	
	Aromatic aminotransferases	Phenylpyruvate decarboxylase
YS58-YEp	0.20 ± 0.01	0.06 ± 0.01
YS58(ARO8-58)	0.80 ± 0.02	0.07 ± 0.01
YS58(ARO10-58)	0.19 ± 0.02	0.29 ± 0.01
YS58(YEpAP58)	0.50 ± 0.01	0.14 ± 0.01
YS58(ARO8 + ARO10)	0.85 ± 0.03	0.36 ± 0.02
YS58(A8-A10-A2)	0.83 ± 0.02	0.37 ± 0.01
YS58(G1-A8-A10-A2)	0.98 ± 0.02	0.42 ± 0.02
YS58(G1-A8-A10-A2)-GDH	0.96 ± 0.03	0.43 ± 0.01

^a^YS58-YEp, strain with control vector; YS58(ARO8-58), YS58(ARO10-58) and YS58(YEpAP58), strains with *ARO8*, *ARO10* and *GAP1* expression cassettes respectively; YS58(ARO8 + ARO10), *ARO8* and *ARO10* co-expression strain; YS58(A8-A10-A2), *ARO8 *+ *ARO10 *+ *ADH2* co-expression strain; YS58(G1-A8-A10-A2), strain with *GAP1 *+ *ARO8 *+ *ARO10 *+ *ADH2* co-expression; YS58(G1-A8-A10-A2)-GDH, strain with *GAP1 *+ *ARO8 *+ *ARO10 *+ *ADH2 *+ *GDH2* co-expression cassettes

** Significance of difference P < 0.01

### Fermentation optimization of modularized engineering strain

**Fig. 4:**
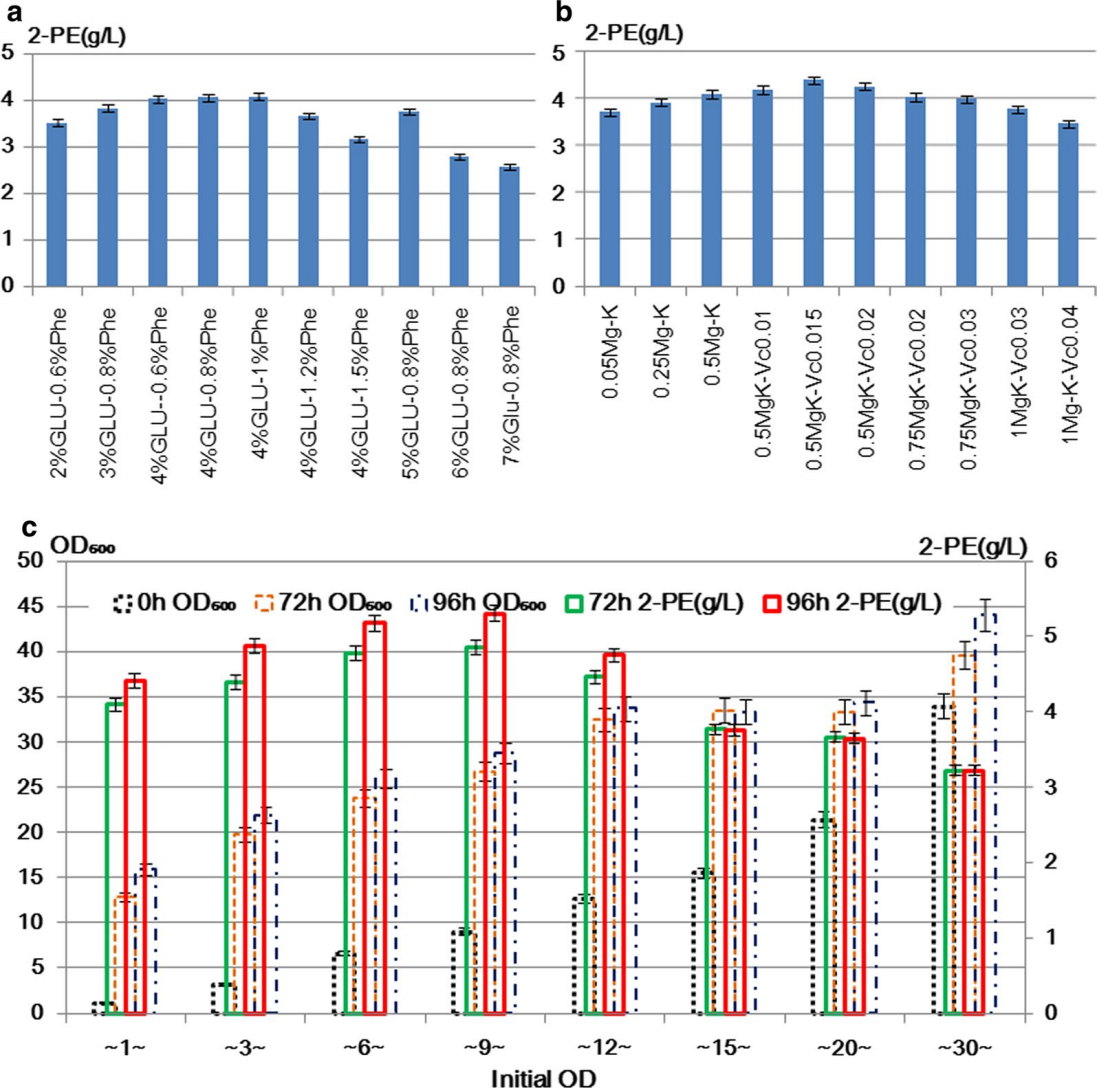
Fermentation conditions optimization of modularized strain YS58(G1-A8-A10-A2)-GDH for 2-PE synthesis. **a** Comparison of the affection of different concentrations of glucose and l-Phe on 2-PE production with initial OD_600_ ~ 1 and 0.5% of magnesium and potassium ions in medium. **b** Comparison of the affection of different concentrations of inorganic salts and ascorbic acid on 2-PE synthesis with 4% of glucose and 0.8% of l-Phe. **c** Affection of inoculum concentrations and cell densities on 2-PE production with 0.5% of magnesium and potassium ions, 4% of glucose and 0.8% of l-Phe

With plasmids increase in yeast cells, the metabolic overload may increase and the medium used in previous study might be innutritious. Hence fermentation conditions optimization was done. The results of 2-PE titers at different concentrations of glucose and l-Phe indicated that less or excessive carbon source and nitrogen substrate were both unfavorable for 2-PE synthesis, the optimum concentration of glucose was about 40 g L^−1^, and the concentration of l-Phe should be less than 10 g L^−1^ (Fig. 4a). Some ions are necessary for cellular metabolism, for example, magnesium is activator for many enzymes, phosphate ion affect redox reaction and other metabolic synthesis that is generally necessary elements for cells. According to the previous reports, magnesium and potassium were necessary ions [10, 35, 45, 46], while zinc and sodium had no significant affection on 2-PE synthesis in *S. cerevisiae* [47]. In our previous study, the medium condition suitable for our other engineering strains for 2-PE synthesis was established [10, 18]. In preliminary experiments of this study, the affection of NaCl on 2-PE synthesis was tested, differently, our results showed that NaCl suppressed 2-PE synthesis in our module strain, not necessary as for strain reported by Tian et al. [48], which might be owing to strains’ differences. The results of affection of inorganic salts on 2-PE synthesis in our study showed that the engineering strain YS58(G1-A8-A10-A2)-GDH produced higher 2-PE when 5 g L^−1^ MgSO_4_·7H_2_O and 5 g L^−1^ KH_2_PO_4_ were contained in the medium (Fig. 4b).

Besides carbon, l-Phe substrate and inorganic salts, it has been reported that ascorbic acid (Vitamin C, Vc) could improve the redox reaction and suppress the generation of by-products during biotransformation of natural 2-phenylethanol [48]. Considering that the increase of reducing power might promote the third step reaction in Ehrlich pathway, ascorbic acid was added into the fermentation medium in this work. The results showed that appropriate amount of ascorbic acid (0.15 g L^−1^) was indeed helpful to 2-PE synthesis (Fig. 4b).

Moreover, synthesis of aromatic alcohols are also controlled by cell density and morphology. In our work, when cell density was less than OD_600_ 30, 2-PE was subjected to positive feedback regulation (Fig. 4c), which was similar to that in Chen and Fink’s report [49]. The optimum cell densities for efficient 2-PE synthesis were about OD_600_ 25–30 with OD_600_ 6–9 of inoculum (Fig. 4c). When cell density was too high, 2-PE yield decreased. Especially when cell density was more than OD_600_ 40, the strain had a greater drop in 2-PE yield which might be caused by changed morphology of the yeast cells under crowded environment, indicating that this strain was not suitable for high cell density fermentation.

**Fig. 5:**
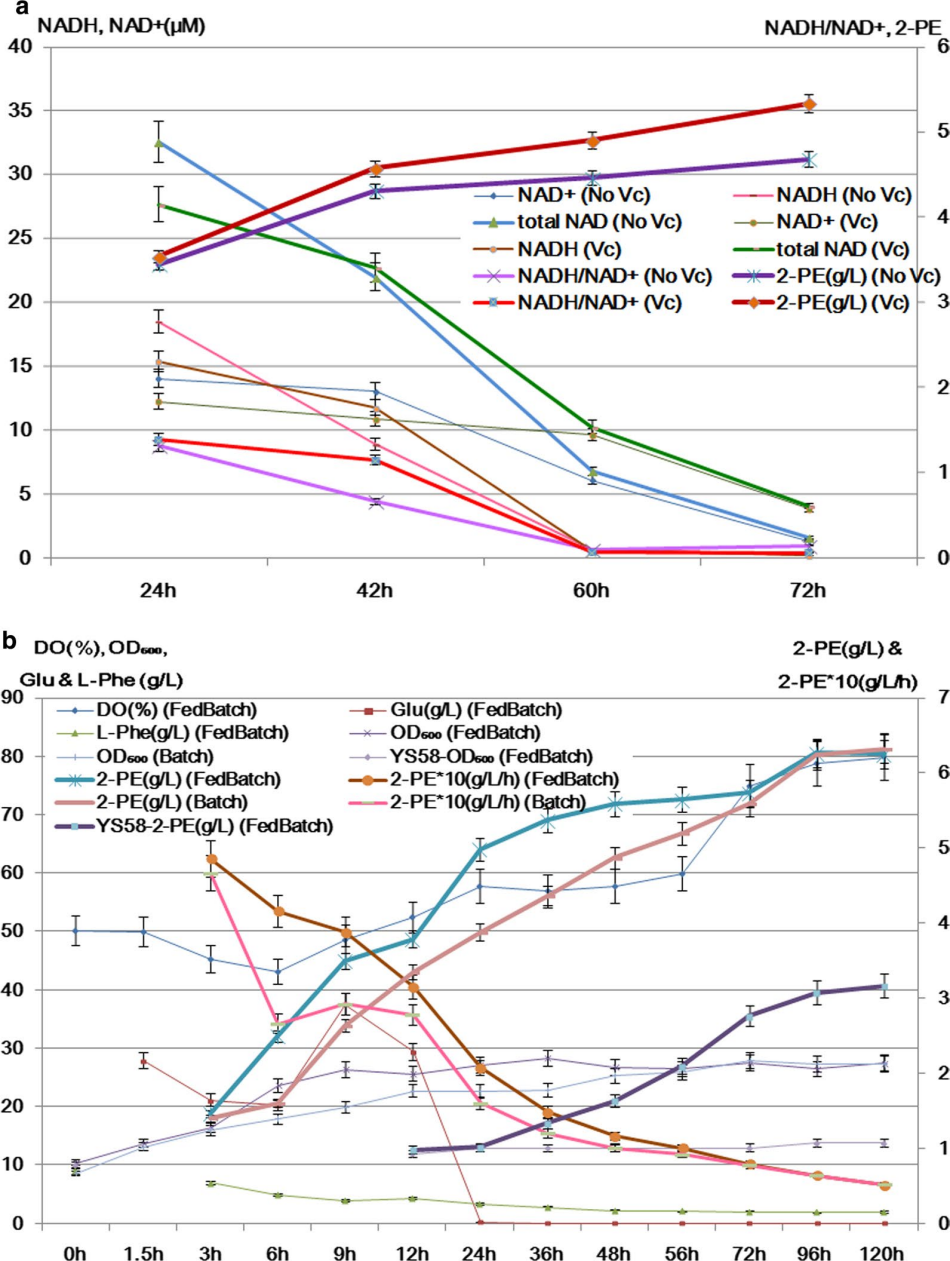
Fermentation of YS58(G1-A8-A10-A2)-GDH strain. **a** Comparison of redox levels and 2-PE titers in flask fermentation with initial OD_600_ ~ 6 in optimum medium. **b** Comparison of 2-PE production in 5 L fermenter of single-batch and fed-batch fermentation with initial OD_600_ ~ 9 in optimum medium. For fed-batch fermentation, glucose (Feed 1) was fed from 3 to 11 h, and l-Phe (Feed 2) was fed from 8 to 11 h

Under the optimum condition, modularized YS58(G1-A8-A10-A2)-GDH strain was fermented in flask and redox reaction was tested. Redox level reflects metabolic activity relative to cell growth, biosynthesis and so on [50, 51]. NAD^+^, NADH and NADH/NAD^+^ ratio are crucial indexes of redox reaction. The levels of NAD^+^ and NADH content control metabolic flux in cells [52]. The ratio between NAD^+^ and NADH regulate redox balance [50, 51]. The results showed that both the content of NAD^+^ and NADH in the module yeast strain with or without ascorbic acid addition decreased during the fermentation, however, adding ascorbic acid in the culture led to higher NADH/NAD^+^ ratio during the period from 24 to 60 h of fermentation, reaching 1.7 folds higher than that without ascorbic acid at 42 h, which might enhance the yeast metabolic activity resulting in increased 2-PE titer (Fig. 5a).

Based on the above test, batch and fed-batch fermentation of modularized YS58(G1-A8-A10-A2)-GDH strain in 5 L fermenter were carried out for further comparison of 2-PE synthesis (Fig. 5b). Oxygen could inhibit the activity of alcohol acetyltransferase that catalyzes 2-phenylethanol to its ester 2-phenylethylacetate, hence, higher shaking speed was helpful to 2-PE accumulation avoid of its esterification [53]. In our study, when ventilatory capacity was about 5 L min^−1^ with 400–500 rpm shaking speed (~ 43–55% dissolved oxygen, DO) during the earlier 22 h and with 300 rpm shaking speed (~ 60% DO) during 22–60 h, 2-PE titers in 5 L of batch fermenter were about 6% higher than that in flask, indicating that raising aeration appropriately was helpful to 2-PE synthesis. Similar to flask fermentation, 2-PE titers during single-batch fermentation increased linearly before 96 h and reached a plateau at 96 h (Fig. 5b). During fed-batch fermentation, 2-PE productivity during 24–56 h was higher than that of single-batch fermentation, while 2-PE production after 72 h was similar to that of batch fermentation. The increased 29% of 2-PE titer and productivity at 24 h might mainly be due to higher biomass caused by glucose feeding (Fig. 5b). Feeding of glucose could speed up yeast cells growth, when the biomass reached the maximum, more feeding had no positive role on 2-PE yield. There were no significant differences among three feeding ways of l-Phe, initial l-Phe content (7 g L^−1^) with feeding, initial l-Phe content (9 g L^−1^) without or with feeding of l-Phe during the fermentation, therefore, enough content of l-Phe at beginning was appropriate. The differences of 2-PE production between batch and fed-batch were gradually minified with the biomass in single-batch fermentation increased, 2-PE titers were very close to each other during 72–120 h (Fig. 5b). When the fermentation was finished, 2-PE titer was ~ 6.3 g L^−1^ whose with conversation ratio 95% (Fig. 5b).

Although there was no difference of 2-PE production between single-batch and fed-batch after 72 h fermentation, the significantly enhanced 2-PE synthetic rate before 24 h was very favorable to fed-batch with product extraction. Since 2-PE titers at 12 and 24 h (3.79 and 4.99 g L^−1^) during fed-batch were very close to that at 24 and 48 h (3.88 and 4.88 g L^−1^) during single-batch, when in situ product adsorption was combined in later study, shortening half a day or 1 day was economic for the whole production craft.

Compared to other engineering strains, the module strain YS58(G1-A8-A10-A2)-GDH in our study presented efficient 2-PE synthesis ability. 2-PE titer in metabolic engineering *S. cerevisiae* with *ARO9 *+ *ARO10 *+ *ARO80* co-expression strain and *ALD3* deletion reported by Kim et al. reached 4.8 g L^−1^ at 195 h of fermentation [17], in our study, the module strain produced 4.99 g L^−1^ of 2-PE at 24 h under fed-batch condition, and produced 4.88 g L^−1^ of 2-PE at 48 h under single-batch condition (Fig. 5b), the reduced fermentation time and higher synthetic rate were advantageous for large-scale production later. Genetic engineered *S. cerevisiae* with *ARO8 *+ *ARO10* co-expression reported by Yin et al. produced 2.6 g L^−1^ of 2-PE at 60 h under fed-batch fermentation, higher 36.8% than that of control strain [41], in our study, 2-PE titer at 56 h under fed-batch condition was 5.65 g L^−1^, 2.7 folds higher than that of control (Fig. 5b). 2-PE yield of our module strain at 56 h was 2.2 folds higher than that of engineered yeast of Yin et al. [41], indicating that the module design and promoter strategy in our study were helpful for efficient 2-PE bio-synthesis.

## Conclusions

In this study, metabolic modularization and promoter strategy was feasible for 2-phenylethanol synthesis efficiently. Due to multi-proteins’ over-expression, including permease for l-Phe transportation, catalytic enzymes transaminase, decarboxylase and dehydrogenase for 2-PE synthesis via Ehrlich pathway and glutamate dehydrogenase for re-supplying another substrate and relieving repression, the produced 2-PE in the module strain increased to ~ 6.3 g L^−1^, whose higher 2-PE yield had potentiality for application. The enhanced productivity and shortened fermentation time by glucose feeding would be important for later two phases fermentation.

## Methods

### Microorganisms, plasmids and cultivations

Strains used in this study are shown in Table 2. *Escherichia coli.* DH5α(*sup*E44 Δ*lac*U169(*φ*80*lac*ZΔM15) *hsd*R17 *rec*Al *end*Al *gyr*A96 *thi*-1 *rel*Al) was used for harboring the recombined plasmids. *S. cerevisiae* YS58 (*MATα flo1 ura3*-*52 leu2*-*3112 his4*-*519 trp1*-*789*) was kindly given by Prof. Teunissen, which was derived from diploid yeast YS60 [54], and was used as the donor for *ARO8*, *ARO10*, *ADH2* and *GDH2* gene. Coding sequences of *GAP1, ARO8*, *GDH2*, *ARO10* and *ADH2* cloned in the recombined plasmids were sequenced before transforming into the yeast strains.

**Table 2:** Plasmids and strains used in this study

Plasmids or strains	Descriptions	References or sources
Plasmids		
pMP1	Plasmid containing promoter *PGK1*	[55]
pYCUP	Plasmid containing *CUP1*	[56]
YEpAP58	Plasmid containing *GAP1*	[10]
YEpKA	Plasmid containing *kanMX*	[10]
YEp-KUA	Plasmid containing promoter UAS_TEF-RG-_*ADH1p*	[43]
YEp-KUT	Plasmid containing promoter UAS_TEF-RP-_*TEF1p*	[43]
MP1-ARO8	Plasmid containing *ARO8* with promoter *PGK1p*	This study
KUT-ARO10	Plasmid containing *ARO10* with promoter UAS_TEF-RP-_*TEF1p*	This study
KUA-ADH2	Plasmid containing *ADH2* with promoter UAS_TEF-RG-_*ADH1p*	This study
pGAP1-ARO8	Plasmid containing expression cassettes of *GAP1* and *ARO8*	This study
Y-ARO10-ADH2	Plasmid containing expression cassettes of *ARO10* and *ADH2*	This study
pYCUP-GDH2	Plasmid containing expression cassettes of *CUP1* and *GDH2*	This study
Strains		
*E. coli.* DH5α	*sup*E44 Δ*lac*U169(*φ*80*lac*ZΔM15) *hsd*R17 *rec*Al *end*Al *gyr*A96 *thi-*1 *rel*Al	Stratagene
*S. cerevisiae* YS58	*MATα flo1 ura3-52 leu2-3*, *112 his4-519 trp1-789*	[54]
YS58-YEp	Recombined yeast strain with selection marker gene *URA3*	[18]
YS58(YEpAP58)	Recombined yeast strain with *GAP1* expression cassette	[10]
YS58(ARO8-58)	Recombined yeast strain with *ARO8* expression cassette	[18]
YS58(ARO10-58)	Recombined yeast strain with *ARO10* expression cassette	[18]
YS58(ADH2)	Recombined yeast strain with UAS_TEF-RG-_*ADH1p*-*ADH2*	This study
YS58(GDH2)	Recombined yeast strain with *GDH2* expression cassette	This study
YS58(YEp-YKA)	Recombined yeast strain with marker genes *URA3* and *kanMX*	This study
YS58(ARO8 + ARO10)	Recombined yeast strain with *ARO8* + *ARO10* expression	This study
YS58(A8-A10-A2)	Recombined yeast strain with *ARO8* + *ARO10* + *ADH2*	This study
YS58(G1-A8-A10-A2)	Recombined yeast with *GAP1* + *ARO8* + *ARO10* + *ADH2*	This study
YS58(YEp-YKA-YCUP)	Recombined yeast with marker genes *URA3*, *kanMX* and *CUP1*	This study
YS58(G1-A8-A10-A2)-GDH	Recombined yeast with *GAP1* + *ARO8* + *ARO10* + *ADH2* *+* *GDH2*	This study

Plasmids used in this study are shown in Table 2. The PCR-amplified *ARO8* ORF from YS58 genomic DNA was cloned into the *Eco*RI/*Xba*I sites of pMP1 [55] for plasmid MP1-ARO8 construction. The fragment of *PGK1* promoter and *ARO8* ORF from plasmid MP1-ARO8 was cloned into the *Kas*I site of YEpAP58 [10] for constructing plasmid pGAP1-ARO8. *ARO10* ORF was cloned into *Xma*I/*Sph*I sites of YEp-KUT [43] generating plasmid KUT-ARO10. *ADH2* ORF was cloned into *Xma*I/*Eco*RI sites of YEp-KUA [43] resulting in plasmid KUA-ADH2. Fragment UAS_-TEF-RP_-*TEF1* promoter and *ARO10* from plasmid KUT-ARO10 was cloned into *Aat*II site of plasmid KUA-ADH2 for plasmid Y-ARO10-ADH2 construction. *GDH2* gene was cloned into *Kpn*I/*Bgl*II of plasmid pYCUP [56] resulting in plasmid pYCUP-GDH2. Plasmids pGAP1-ARO8, Y-ARO10-ADH2 and pYCUP-GDH2 were used for sequencing and transformation. Primer sequences used for plasmid construction are shown in Table 3.

**Table 3:** Primers used in this study

Primers	Sequences (5′-3′)^a^	Purposes
P1	CCGGAATTC ATGACTTTACCTGAATCAA	P1/P2: for PCR of *ARO8*
P2	CTAGTCTAGAATAAAGTTGTACTCTTAATG	
P3	CCCGGCGCCTCGAGGTCGACGGTATC	P3/P4: for PCR of *PGK1p *+ *ARO8*
P4	CTAGGGCGCCATAAAGTTGTACTCTTAATG	
P5	TCCCCCCGGG ATGGCACCTGTTACAATTGAA	P5/P6: for PCR of *ARO10*
P6	ACATGCATGC ACTCTGTGGTAGTGGTAAAA	
P7	TCCCCCCGGGATGTCTATTCCAGAAACTCA	P7/P8: for PCR of *ADH2*
P8	CCGGAATTCTGTCTACAGTTTAGAGGAAT	
P9	CCCGACGTCTTTCGTCTTCAA	P9/P10: for PCR of UAS_TEF-RP-_*TEF1p* +*ARO10*
P10	CCCGACGTCACTCTGTGGTAGTGGTAAAA	
P11	CGGGGTACCCCGCAAGTAATACTTACAGCACT	P11/P12: for PCR of *GDH2*
P12	GGAAGATCTTCCGCTGAGAAGAACATACAC	
P13	GGCATTGGCACTCATGACCT	P13/P14: for PCR of part of *CUP1 *+ *GDH2* P15/P16: for PCR of UAS_TEF-RG-_*ADH1p* +*ADH2* P17/P18: for PCR of *ADH1p* +*GAP1 *+ *ADH1t*
P14 P15 P16 P17 P18	CTTTGGAGCATGGTAAGGATACC GCATGCCCGCGGCCACACA TGTCTACAGTTTAGAGGAAT TTATTCTTTCCTCTG GCCTCGACTGAAGGCTAGG	
P19	GTCATCACTATTGGTAACG	P19/P20: for qRT-PCR of *ACT1*
P20	GGAGTTGTAAGTAGTTTGG	
P21	GCTGTTATCTTCCCTATTTCG	P21/P22: for qRT-PCR of *GAP1*
P22	GTAGCACAACCAACCATT	
P23	GCCGCAACAGATGGATATTT	P23/P24: for qRT-PCR of *ARO9*
P24	GCATAGGCGATGGTGAGTCT	
P25	TACCAAGATTATCCACGATT	P25/P26: for qRT-PCR of *ARO8*
P26	AATGTGCCTCAACTAAGAT	
P27	CGCTTACAAGCGATTCACCA	P27/P28: for qRT-PCR of *GDH2*
P28	GATGTCATCATCCTTAACAT	
P29	AACGCTCACATCAATGGT	P29/P30: for qRT-PCR of *ARO10*
P30	ATGGTGCTCAGTTCTTGG	
P31	CTGCTGGTGGTCTAGGTTC	P31/P32: for qRT-PCR of *ADH2*
P32	CCGAGCGAGGTAAACAATTC	

^a^The underlined font in primers 1–12 represents the sequences for restriction enzyme *Eco*RI, *Xba*I, *Kas*I, *Kas*I, *Xma*I, *Sph*I, *Xma*I, *Eco*RI, *Aat*II, *Aat*II, *Kpn*I and *Bgl*II respectively

*Escherichia coli* strain was grown at 37 °C in Luria–Bertani medium [57] supplemented with ampicillin (100 mg L^−1^) when necessary. Some yeast strains such as YS58(YEp-YKA), YS58(ARO8 + ARO10), YS58(A8-A10-A2) and YS58(G1-A8-A10-A2) were screened from a selective synthetic complete (SC) medium (10 g L^−1^ glucose, 6.7 g L^−1^ yeast nitrogen base, 40 mg L^−1^ histidine, 40 mg L^−1^ leucine, 40 mg L^−1^ tryptophan) with no uracil but adding 200 mg L^−1^ geneticin 418 (G418). Strains YS58(YEp-YKA-YCUP) and YS58(G1-A8-A10-A2)-GDH were screened from YPD medium (10 g L^−1^ yeast extract, 20 g L^−1^ peptone and 20 g L^−1^ glucose) with 8 and 11 mM copper sulfate (CuSO_4_) respectively. YS58(ADH2) was screened on YPD with 200 mg L^−1^ G418. YS58(GDH2) was screened on YPD with 8 mM CuSO_4_.

The recombinants strains were preserved on selective medium, and were also stabilized by alternating cultivation in non-selective medium YPD for about 20 generations followed by shifting to the selective medium. Then they were verified by PCR. In addition, the strains after fermentation were also analyzed to convince that the plasmids were kept.

The optimum fermentation medium used for 2-PE synthesis contained 40 g L^−1^ glucose, 5 g L^−1^ MgSO_4_·7H_2_O, 5 g L^−1^ KH_2_PO_4_, 0.15 g L^−1^ ascorbic acid and 9 g L^−1^ l-phenylalanine (l-Phe) as a sole nitrogen source. Yeast strains were first grown in YPD medium with 2% inoculation, and cultivated at 30 °C (shaking speed 220 rpm) for 24 h, then inoculated the collected pellets to 40 mL fermentation medium in 250 mL shake flask or to 2.5 L fermentation medium in 5 L fermenter with initial OD_600_ of ~ 6 or ~ 9, and cultivated at 30 °C in flask with shaking at 220 rpm or in batch/fed-batch fermenter with 300–500 rpm, 4–5 L min^−1^ of ventilatory capacity, 40–80% of dissolved oxygen (DO) and 0.05 MPa of tank pressure. Medium for fed-batch: 500 g L^−1^ glucose (Feed 1) and 30 g L^−1^ l-Phe (Feed 2) with the same concentration of MgSO_4_/KH_2_PO_4_/ascorbic acid as above. In tube tests, the yeast strains from YPD medium were inoculated into 5 mL of fermentation medium with initial OD_600_ of ~ 1 or 1–30 according to experimental design, then shook at 220 rpm and 30 °C for 96 h. 2-PE titers and other indexes at different time points were analyzed and compared.

### Quantitative RT-PCR (qRT-PCR) and analytical methods

#### Quantitative RT-PCR

Module strain was cultured under optimum conditions, and samples were collected at 24 h. Total RNA was isolated from yeast cells and the amounts of mRNAs of *GAP1*, *ARO8*, *ARO9*, *GDH2*, *ARO10* and *ADH2* were determined by qRT-PCR. Total RNA (1 μg) was first incubated in DNA removing mixture (10 μL) containing DNase (TIANGRN Biotech Co., Ltd, Beijing, China) at 42 °C for 3 min. RNA was then subjected to reverse transcription in 20 μL reaction mixture containing Quant Reverse Transcriptase (TIANGRN Biotech Co., Ltd) and 0.1 mg oligo-dT (M-biotech, Inc.) at 42 °C for 60 min. Reaction mixture of 50 ng cDNA, 10 μL SuperReal PreMix Plus (with SYBR Green I) and gene-specific primers (Table 3) were subjected to qPCR reaction. PCR was performed with 40 cycles of 95 °C for 10 s, 58 °C for 20 s, and 72 °C for 20 s using Roche LightCylcer 96 real-time PCR system (Roche Diagnostics). Expression data were processed by the second-derivative maximum method of LightCycler 96 software SW1.1. Delta cycle threshold (Δ*C*_T_) values were calculated by the *C*_T_s of the target genes minus *C*_T_ of the *ACT1* gene as a housekeeping gene. ΔΔ*C*_T_ values were calculated by Δ*C*_T_ values of the experimental samples −Δ*C*_T_ of the wild-type sample. Fold changes were calculated using the 2^−ΔΔ*C*T^ method [58]. Enhanced folds of transcription levels were the ratios of gene/*ACT1* values of transformants divided by that of control strain YS58-YEp.

#### Analysis of enzyme activities

Crude enzyme extraction: cell pellets from 2 mL yeast culture were first frozen in 1 mL of potassium phosphate (10 mM) with 2 mM EDTA (pH7.5) overnight, then re-suspended in 0.4 mL of potassium phosphate (100 mM) with 0.1 mM EDTA (pH6.5), after vortex oscillation with 50 mg of glass beads (diameter 0.5 mm) for 4 min, supernatant crude enzyme was obtained. The protein content was determined by Bradford method using Coomassie plus protein assay reagent with bovine serum albumin (BSA) as the standard protein [59]. Test for aromatic aminotransferases: The reaction mixtures (1 mL) contained potassium phosphate buffer (0.1 mM, pH 8.0), pyridoxalphosphate (0.1 mM), l-phenylalanine (1 mM), pyruvate (10 mM) as amino acceptor and cell free extract (0.2–0.6 mg protein). The reactions were started by the addition of l-phenylalanine. Two parallel reactions of aminotransferase activities were stopped after 2 and 10 min at 30 °C by the addition of 1 mL of 1 M NaOH on ice. Activity was measured by monitoring the increment of phenylpyruvate, the reaction product of the phenylalanine aminotransferase at 320 nm, ε = 1.7500 × 10^4^ cm^2^ mol^−1^ [12, 60]. Enzyme activities for phenylpyruvate decarboxylase: The reaction mixtures (1 mL) contained potassium phosphate buffer (70 mM, pH 7.0), NAD^+^ (2 mM), thiamine pyrophosphate (0.2 mM), yeast acetaldehyde dehydrogenase (0.35 U, dissolved in 1 mM dithiothreitol)), phenylpyruvic acid (2 mM) and cell free extract (~ 0.5 mg protein). The reactions were started by the addition of phenylpyruvic acid. Activity was measured by monitoring the reduction of NAD^+^ at 340 nm, ε = 6.2 × 10^3^ cm^2^ mol^−1^ [61]. International unit (IU) was adopted for the above two enzyme activities: the amount of enzyme required to convert 1 μmol of substrate to product per minute under a designated condition.

#### NADH/NAD^+^

1 mL of yeast culture was centrifuged and washed with 1 mL cold phosphate buffer. Homogenize the pellet with either 100 µL NAD extraction buffer (BioAssay Systems Co., Ltd.) for NAD determination or 100 µL NADH extraction buffer (BioAssay Systems Co., Ltd.) for NADH determination. Heat extracts at 60 °C for 5 min and then add 20 µL Assay Buffer (BioAssay Systems Co., Ltd.) and 100 µL of the opposite extraction buffer to neutralize the extracts. Briefly vortex and spin the samples down at 14,000 rpm for 5 min. A small portion (40 µL) of the supernatant was immediately mixed with 80 µL of prepare working reagent (60 µL Assay Buffer, 1 µL Enzyme A, 1 µL Enzyme B, 14 µL Lactate and 14 µL MTT) from the EnzyChrom™ NAD+/NADH Assay Kit (E2ND-100, BioAssay Systems Co., Ltd.). Optical density (OD_0_) for time “zero” at 565 nm and OD_15_ after 15 min of incubation at room temperature were detected. ∆OD for each sample by subtracting OD_0_ from OD_15_ was then used to calculate NAD(H) concentration [62, 63].

l-Phe contents and 2-PE titers in the culture medium were detected using Agilent 1260 high performance liquid chromatography system equipped with C-18 column and DAD-detector at 254 and 260 nm respectively (Agilent Technologies). Methanol (50%) and water (50%) were pumped as mobile phase at 1.0 mL min^−1^. All data are presented as the averages of the results of three independent experiments. Error bars show standard deviations.

### Nucleotide sequences accession numbers

Accession numbers of *GAP1*, *ARO8*, *GDH2*, *ARO10* and *ADH2* of YS58 are the same as that of *S. cerevisiae* S288C on National Center for Biotechnology Information (NCBI), and the Accession Numbers are respective NM_001179829 (*GAP1*), NM_001181067.1 (*ARO8*), NM_001180275 (*GDH2*), NM_001180688.3 (*ARO10*) and NM_001182812 (*ADH2*).

## Abbreviations

l-Phe: l-phenylalanine
2-PE: 2-phenylethanol
G418: geneticin 418
*ADH1p*: *ADH1* promoter
*PGK1p*: *PGK1* promoter
UAS_-TEF-RP_-*TEF1p*: UAS_-TEF-RP_-*TEF1* promoter
UAS_-TEF-RG_-*ADH1p*: UAS_-TEF-RG_-*ADH1* promoter
IU: international unit
DO: dissolved oxygen
